# Childhood obesity in Mexico: Influencing factors and prevention strategies

**DOI:** 10.3389/fpubh.2022.949893

**Published:** 2022-08-18

**Authors:** Teresa Shamah-Levy, Lucia Cuevas-Nasu, Elsa B. Gaona-Pineda, Danae G. Valenzuela-Bravo, Ignacio Méndez Gómez-Humarán, Marco A. Ávila-Arcos

**Affiliations:** ^1^Center for Evaluation and Survey Research, National Institute of Public Health, Cuernavaca, Mexico; ^2^Center for Mathematics Research, Aguascalientes Unit, Aguascalientes, Mexico

**Keywords:** overweight, obesity, body mass index, prevention strategies, Mexico

## Abstract

**Background:**

Overweight and obesity in school-age children, in Mexico as in other countries around the world, is a rapidly increasing public health problem within recent years, with important consequences for the future health of the population. Various national strategies at the individual and community level have been established to prevent these conditions, but none have yet succeeded.

**Objective:**

To describe factors which influence overweight and obesity in school-age children five to 11 years old in Mexico, and national strategies for the prevention and management of these conditions.

**Methods:**

The data herein is derived from six National Health and Nutrition Surveys in Mexico: 2006, 2012, 2016, 2018, 2020, and 2021. They include a total of 45,216 school-age children with complete anthropometric data (weight/height) distributed over 84 pseudo-panels defined by age, wellbeing condition class (WCC), gender, and type of locality of residence. The indicators calculated were overweight and obesity by body mass index according to World Health Organization guidelines. Predictors are food consumption indicators in five groups.

**Results:**

The prevalence of overweight and obesity showed a positive linear trend (*p* < 0.001), with average annual increases of 0.41%. Increases in fruit consumption reduced the prevalence of these conditions by 6.6% (*p* = 0.01) and vegetable consumption reduced this by 8.3%.

**Conclusions:**

Overweight and obesity in school-age children is a growing problem with serious repercussions for future life. New strategies are needed which focus on involving food systems, which translates to healthy and sustainable diets.

## Introduction

In Mexico, overweight and obesity has been recognized as a public health problem. This is thanks to the National Nutrition Surveys of 1988 and 1999 and the first National Nutrition and Health Survey (ENSANUT for its Spanish acronym) in 2006, which recorded significant increases in the prevalence of these conditions in women and children (1). In school-age children, the prevalence of overweight and obesity was 34.8% in 2006, 34.4% in 2012, 33.4% in 2016, 35.6% in 2018, 38.2% in 2020, and up to 37.3% in the year 2021 (2–4).

Over time, public policies have been established to address population health and nutrition problems. The principal programs created were *Prospera* (previously *Oportunidades*, then *Progresa*), the social milk supply program *Liconsa*, and the school lunch program (*Desayunos Escolares*). However, their objectives were historically based in achieving food access for the most vulnerable groups, and they were not designed from their beginnings to address the growing epidemic of overweight and obesity in Mexico (5).

Given the large burden that overweight and obesity can represent to health systems, the Organization for Economic Cooperation and Development (OECD) countries are expected to allocate 5.4% of their total health budget to treating conditions derived from obesity: 311 billion USD purchasing power equivalent per year. Obesity in school-age children has been associate with poorer academic performance (13%) and in later life stages it may have implications for human capital and socioeconomic level (6). In 2010, the Ministry of Health of Mexico developed and implemented the National Agreement for Nutritional Health (ANSA for its Spanish acronym), which promoted the development of healthy life skills (7). From the ANSA were born the General Guidelines for the Sale and Distribution of Prepared and Processed Food and Drinks in Schools within the National Educational System, with the goal to establish technical nutritional criteria to regulate the preparation, sale, and distribution of prepared and processed food and drinks appropriate for healthy eating within the public and private schools of the National Educational System (8). Later, in the year 2013, the Ministry of Health of Mexico implemented the National Strategy for the Prevention and Management of Overweight, Obesity and Diabetes, which aimed to slow the growing prevalence of overweight and obesity, as well as chronic diseases such as type two diabetes (9).

Furthermore, given that sugar-sweetened beverages are well understood as a casual factor of overweight and obesity, the year 2014 saw the implementation of a tax of one Mexican peso per liter on beverages with added sugars, as well as on food items with an energy content ≥275 calories per 100 g (10). In 2020, implementation began of the frontal warning label on packaged food items, as well as restrictions on the use of characters used on food packages to target marketing at children (11).

The present article describes factors influencing overweight and obesity in school-age children from 5 to 11 years of age in Mexico between the years 2006 and 2021, and national strategies for the prevention of these conditions.

## Methods

We analyzed the information from ENSANUT surveys applied in 2006, 2012, 2016, 2018-19, 2020, and 2021. ENSANUTs have a probabilistic, stratified design by conglomerates; details on ENSANUT sampling procedures have been published previously (12) and ENSANUT databases and questionnaires may be retrieved in the webpage https://ensanut.insp.mx/ (13). For the present analysis, the population of interest was all children from 5 to 11 years of age selected in each year of the survey. The sample size in each survey was:14,990 school-age children in 2006; 16,351 in 2012; 3,179 in 2016; 6,183 in 2018–19, 1,944 in 2020, and 2,569 in 2021.

### Variables

#### Nutritional status

All participants were weighed and measured by staff trained in internationally standardized methods (14, 15). Weight was measured with a SECA brand electronic scale produced in Germany, and height was measured with a SECA brand wall stadiometer produced in Germany. Using the tools previously mentioned, Z-score of body mass index (BMI) was calculated (BMI = kg/m^2^) by age and sex. Using the standard references of the World Health Organization (WHO) (16), overweight (OW) was defined as a Z-score >+1 and up to +2 standard deviations, while obesity (OB) was defined as >+2 and up to +5.5 standard deviations.

#### Diet

Using information from a semi-quantitative food consumption frequency questionnaire, we classified food items in the following groups: **fruits** (peach/nectarine, strawberry, guava, jicama, lime, mango, apple/pear, cantaloupe/watermelon, orange/tangerine, papaya, pineapple, banana, grapefruit, grapes), **vegetables** (avocado, broccoli/cauliflower, zucchini, onion-for example in salads, street snacks or fast foods–chayote, green chili, dried chili, cabbage, green beans, corn, leafy greens, tomato, lettuce, cactus, cucumber, frozen vegetables such as peas, carrot, broccoli, cauliflower, or green beans, canned vegetables such as peas, carrots, mushrooms, and green beans and carrot), **plain water**, **snack foods**, **sweets and desserts** [chocolate, dairy-based or imitation desserts, candy (hard candies, lollipops), chili-coated candies, fried items (all types, including Japanese-style peanuts), candied or dried fruits, ice cream and dairy-based popsicles, water-based popsicles and shaved ice, fruits stored in nectar, gelatin, flan, marshmallow lollipops, microwave or movie popcorn (all types except caramel-covered), cake, pie], and **sugar-sweetened beverages** [natural fruit water with sugar added, corn-based *atole* with water, commercial flavored drinks with sugar, coffee with sugar added and with or without milk, natural juices with sugar added, commercial fruit nectars or pulp with sugar added, soda, tea with sugar added, dairy-based drink with lactobacillus, corn-based *atole* with milk, milk with added sugar or chocolate, prepared flavored milk (chocolate or other), drinkable whole milk yogurt with fruit, natural drinkable whole milk yogurt]. Consumers were defined as those school-age children who reported consumption of the food group of interest at least 3 days a week, in a quantity of at least 10 g. In the case of fruit, vegetables, and plain water, consumers were defined as those who consumed at least 10 grams seven days a week. Furthermore, derived from a database of nutritional composition (17, 18), we estimated the consumption of total energy and fiber per day per child.

#### Age

We classified age in years completed.

#### Sex

We classified sex as masculine or feminine.

#### Residence locality type

We classified localities with <2,500 inhabitants as rural (R), and those with ≥2,500 inhabitants as urban (U).

#### Wellbeing condition index

We constructed an index of wellbeing conditions based on housing characteristics and household goods and services using principal component analysis. The first component accumulated approximately 50% of the variance across all surveys. The WBI was classified in categories (WCC) using distribution tertiles as cut-off points: low, medium, and high.

### Statistical analysis

From the series of six ENSANUT surveys, which are independent transversal samples, we created a temporary follow-up framework with grouped data in pseudo-panel (19, 20), using the variables age, sex, wellbeing level, and residence locality type (rural or urban). For each pseudo-panel group, we calculated prevalence of overweight and obesity, prevalence of consumption of each food groups, averages of consumption of recommended and not recommended food groups, and averages of consumption of total energy and fiber for each temporary follow-up group. We constructed a longitudinal model using pseudo-panel data, through a mixed model with estimation of random effects for maximum probability. The model allowed establishment of a lineal trend in the prevalence of obesity as a function of time (survey), consumption of fruits, vegetables, snack foods, and sugar-sweetened beverages. Age group, sex, wellbeing level, and residency locality type were used as adjustment variables.

## Results

Table 1 shows the distribution of the main characteristics of the study population by survey year. The distribution by sex has been homogenous across all years, at 50% of each sex. Over 70% of participants have resided in urban localities, and in four of the six surveys the predominant wellbeing level was classified as low.

**Table 1:** Descriptive characteristics of the study population.

**Variable**	**2006**				**2012**				**2016**				**2018**				**2020**				**2021**			
	***n***	**N (thousands)**	**Percent**	**95% CI**	***n***	**N (thousands)**	**Percent**	**95% CI**	***n***	**N (thousands)**	**Percent**	**95% CI**	***n***	**N (thousands)**	**Percent**	**95% CI**	***n***	**N (thousands)**	**Percent**	**95% CI**	***n***	**N (thousands)**	**Percent**	**95% CI**
**Age**																								
5	1,956	2,219.8	14.1	(13.14, 15.2)	2,232	2,250.5	13.7	(12.86, 14.56)	464	2,234.9	14.2	(12.23, 16.33)	857	1,397.5	12.9	(11.62, 14.26)	280	1,907.3	12.4	(10.79, 14.22)	365	2,118.3	13.5	(11.88, 15.33)
6	1,976	2,139.4	13.6	(12.69, 14.62)	2,343	2,290.9	13.9	(13.15, 14.75)	451	2,120.9	13.4	(11.76, 15.3)	903	1,448.7	13.4	(12.21, 14.6)	352	2,367.1	15.39	(13.86, 17.06)	394	2,170.3	13.9	(12.49, 15.33)
7	1,964	1,944.0	12.4	(11.62, 13.18)	2,438	2,331.6	14.2	(13.43, 14.96)	472	2,363.8	15.0	(12.11, 18.36)	964	1,553.9	14.3	(13.07, 15.68)	335	2,262.9	14.71	(12.99, 16.63)	375	2,128.0	13.6	(11.89, 15.46)
8	2,170	2,142.1	13.6	(12.85, 14.48)	2,489	2,381.8	14.5	(13.66, 15.35)	527	2,464.6	15.6	(13.62, 17.82)	984	1,635.8	15.1	(13.8, 16.46)	331	2,160.7	14.05	(12.5, 15.75)	375	2,102.7	13.4	(11.73, 15.31)
9	2,283	2,425.3	15.5	(14.48, 16.47)	2,505	2,330.5	14.2	(13.39, 14.99)	454	2,344.8	14.9	(12.32, 17.8)	951	1,661.9	15.3	(13.8, 16.97)	344	2,258.5	14.69	(13.01, 16.53)	418	2,329.0	14.9	(13.35, 16.52)
10	2,353	2,450.6	15.6	(14.75, 16.5)	2,095	2,265.5	13.8	(12.97, 14.63)	414	2,176.6	13.8	(11.53, 16.4)	769	1,605.8	14.8	(13.3, 16.46)	140	2,038.5	13.25	(11.15, 15.69)	331	2,523.8	16.1	(14.11, 18.32)
11	2,288	2,380.1	15.2	(14.3, 16.06)	2,249	2,593.4	15.8	(14.95, 16.63)	397	2,086.1	13.2	(11.28, 15.41)	755	1,542.3	14.2	(12.8, 15.77)	162	2,384.8	15.51	(13.33, 17.96)	311	2,300.2	14.7	(12.65, 16.97)
**Sex**																								
Female	7,503	7,888.8	50.2	(48.5, 51.01)	8,156	8,116.7	49.4	(49.47, 51.81)	1,591	7,760.3	49.1	(47.82, 53.89)	3,138	5,450.5	50.3	(47.78, 51.71)	941	7,573.0	49.2	(47.89, 53.63)	1,283	7,547.9	48.2	(48.97, 54.69)
Male	7,487	7,812.5	49.8	(48.99, 51.5)	8,195	8,327.4	50.6	(48.19, 50.53)	1,588	8,031.4	50.9	(46.11, 52.18)	3,045	5,395.2	49.8	(48.29, 52.22)	1,003	7,806.8	50.8	(46.37, 52.11)	1,286	8,124.4	51.8	(45.31, 51.03)
**Area**																								
Urban	10,070	11,299.4	72.0	(69.71, 74.11)	10,126	12,262.9	74.6	(73.47, 75.64)	1,368	11,392.0	72.1	(68.09, 75.86)	3,862	7,847.1	72.4	(70.68, 73.96)	1,400	11,272.5	73.3	(70.18, 76.19)	1,838	11,668.7	74.5	(71.92, 76.84)
Rural	4,920	4,402.0	28.0	(25.89, 30.29)	6,225	4,181.3	25.4	(24.36, 26.53)	1,811	4,399.6	27.9	(24.14, 31.91)	2,321	2,998.7	27.7	(26.04, 29.32)	544	4,107.3	26.7	(23.81, 29.82)	731	4,003.5	25.6	(23.16, 28.08)
**WCC**																								
Low	6,280	6,325.3	40.3	(38.15, 42.46)	6,303	5,233.3	31.8	(30.27, 33.42)	1,227	3,923.2	24.8	(21.38, 28.67)	2,534	4,157.2	38.3	(36.26, 40.44)	789	6,119.2	39.8	(36.08, 43.61)	984	5,765.5	36.8	(33.85, 39.82)
Medium	5,246	5,205.1	33.2	(31.49, 34.85)	5,592	5,502.6	33.5	(32.08, 34.88)	1,132	5,200.5	32.9	(29.61, 36.43)	2,155	3,731.1	34.4	(32.31, 36.56)	610	4,720.7	30.7	(27.87, 33.67)	899	5,234.4	33.4	(30, 36.98)
High	3,464	4,171.0	26.6	(24.69, 28.52)	4,456	5,708.3	34.7	(33.02, 36.45)	820	6,668.0	42.2	(37.3, 47.31)	1,494	2,957.5	27.3	(25.3, 29.33)	545	4,539.9	29.5	(26.61, 32.6)	686	4,672.4	29.8	(26.89, 32.91)

ENSANUT 2006, 2012, 2016, 2018-19, 2020, and 2021. Mexico.

The prevalence of OW and OB, by survey years, and the percentages of consume of each food group are presented in Table 2. OW was primarily observed in the first survey years of 2006 (20.3%) and 2012 (20.0%), and in later years leveled at around 18.0%. On the other hand, OB prevalence was lesser in 2006 (14.7%) and shows a sustained increase over the following survey years until reaching nearly 20.0% in 2021. Across all survey years, average consumption of the food groups sugar-sweetened beverages and snack foods was notably greater than consumption of fruits and vegetables.

**Table 2:** Prevalence of nutritional conditions **(A)** proportion of consumer of food groups and intake of total energy and fiber **(B)**, total energy and fiber for each temporary follow-up group.

**Ensanut**	**Normal (%)**				**Overweight (%)**				**Obesity (%)**					
	***n***	***N***	**Prevalence**	**95% CI**	***n***	***N***	**Prevalence**	**95% CI**	***n***	***N***	**Prevalence**	**95% CI**		
**A**														
2006	9,588	10,204,938	65.0	(63.45, 66.5)	3,056	3,187,226	20.3	(19.16, 21.49)	2,346	2,309,197	14.7	(13.64, 15.85)		
2012	10,709	10,734,574	65.3	(64.08, 66.46)	3,189	3,286,670	20.0	(18.97, 21.05)	2,453	2,422,889	14.7	(13.83, 15.69)		
2016	2,197	10,464,529	66.3	(62.09, 70.2)	551	2,891,763	18.3	(15.45, 21.58)	431	2,435,344	15.4	(12.55, 18.81)		
2018	3,955	6,985,674	64.4	(62.26, 66.5)	1,209	1,969,226	18.2	(16.68, 19.73)	1,019	1,890,871	17.4	(15.83,19.16)		
2020	1,205	9,500,278	61.8	(58.89, 64.57)	388	3,038,218	19.8	(17.71, 21.97)	351	2,841,299	18.5	(16.41, 20.74)		
2021	1,577	9,791,124	62.5	(59.51, 65.35)	510	2,923,987	18.7	(16.7, 20.78)	482	2,957,168	18.9	(16.86, 21.05)		
Total	29,231	57,681,117	64.2	(63.11, 65.29)	8,903	17,297,090	19.3	(18.45, 20.08)	7,082	14,856,768	16.5	(15.73, 17.38)		
**Ensanut**	**Fruits (%)**		**Vegetables (%)**		**Snacks, sweets, and desserts (%)**		**Sweetened beverages (%)**		**Plain water (%)**		**Fiber consumption (%)**		**Total energy consumption (Kcal)**	
**B**														
2006	32.5	(30.57, 34.49)	27.0	(24.96,29.18)	65.2	(62.99, 67.26)	90.1	(88.91, 91.23)	79.4	(77.43, 81.23)	15.79	(15.63, 15.95)	1514.60	(1501.9, 1527.3)
2012	45.1	(40.73, 49.55)	29.8	(25.63,34.31)	74.4	(69.76, 78.62)	90.8	(87.95, 93.08)	75.1	(70.89, 78.82)	19.42	(18.939, 19.89)	1723.27	(1690.6, 1755.9)
2016	48.2	(44.4, 52.02)	45.3	(41.68,48.98)	64.4	(60.88, 67.83)	89.8	(87.37, 91.8)	84.8	(81.55, 87.49)	19.56	(19.23, 19.89)	1612.69	(1589.7, 1635.7)
2018	43.6	(41.44, 45.86)	22.0	(20.22, 23.97)	63.9	(61.88, 65.89)	92.7	(91.57, 93.66)	85.5	(83.57, 87.19)	18.12	(17.9, 18.34)	1607.04	(1591.5, 1622.5)
2020	51.6	(46.92, 56.33)	30.5	(26.11, 35.23)	54.1	(49.03, 59.17)	91.4	(88.18, 93.78)	88.0	(84.08, 91.02)	21.31	(20.56, 22.05)	1714.96	(1669.6, 1760.3)
2021	43.6	(38.66, 48.68)	24.1	(19.68, 29.2)	51.0	(45.92, 56.13)	94.1	(91.3, 95.98)	92.3	(89.73, 94.33)	19.74	(18.99, 20.5)	1646.12	(1598.8, 1693.4)
Total	44.1	(42.46, 45.69)	32.2	(30.71, 33.78)	62.2	(60.64, 63.76)	91.2	(90.2, 92.01)	84.7	(83.43, 85.9)	17.6	(17.49, 17.72)	1581.38	(1572.9, 1589.9)

ENSANUT 2006, 2012, 2016, 2018-19, 2020 and 2021. Mexico.

The longitudinal model of pseudo-panel data (Table 3) showed a positive lineal trend in the prevalence of OW and OB (*p* > 0.001), with average annual increases of 0.41%. We observed that an increase in the proportion of consumers of reduced prevalence of OW and OB by 6.6% (*p* = 0.01), while an increase in vegetable consumers contributed a reduction of 8.3%. Snack foods, sweets and desserts, and sugar-sweetened beverages were not statistically significant contributors in the model (*p* = 0.112 and 0.098, respectively), but seem to show weak associations. Of the adjustment variables, we observed that each year of age completed by participants was associated with an average increase of 2.8% in the prevalence of OB. Girls, as compared to boys, showed a 4.2% lower prevalence of OW and OB (*p* < 0.001), and a lower prevalence of these outcomes by 2.8% was shown for all those residing in rural zones. On the other hand, participants with a medium and high level of wellbeing showed an increased prevalence of obesity prevalence, of 6.6 and 12.9%, respectively (*p* < 0.001 for both).

**Table 3:** Linear mixed model for the contribution of food group intake on overweight + obesity.

			**95% CI**	
**Overweight + obesity**	**Coefficient**	***P*-value**	**95% LB**	**95% UB**
ENSANUT	0.0041	0.000	0.0024	0.0059
Fruit consumption	−0.066	0.010	−0.116	−0.016
Vegetable consumption	−0.083	0.000	−0.128	−0.038
Snacks, candies, and dessert consumption	0.041	0.112	−0.010	0.092
Sugar-sweetened beverage consumption	0.075	0.098	−0.014	0.164
Age (years)	0.028	0.000	0.024	0.032
Sex: female	−0.042	0.000	−0.059	−0.025
Area: rural	−0.028	0.002	−0.045	−0.011
Wellbeing condition: medium	0.066	0.000	0.044	0.088
Wellbeing condition: high	0.129	0.000	0.105	0.153
Constant	−8.226	0.000	−11.769	−4.683

## Discussion

The findings of our study show that overweight and obesity in the school-age population, both on the Mexican and international stage, is a growing problem across the last several decades. These outcomes are associated with a greater percentage of consumers of food and drinks with high energy density, lower consumption of fiber, and structural factors previously reported (21, 22) such as belonging to a higher socioeconomic class, residing in urban localities, as well as with age and sex.

Notably, we observed that a decrease in overweight was reported throughout the survey years, at the expense of increases in obesity, where today these two outcomes show similar behavior. In Mexico, OW and OB together in the year 2006 showed a prevalence of 34.8%; in the year 2020 this rose to 38.2%, where prevalence of OW was 20.2 and 19.6%, respectively, and prevalence of OB was 14.6% and rose four percentage points (2).

The pseudo-panels of the study showed that those food groups considered as protective, such as fruits and vegetables, protected against overweight and obesity in school-age children. On the other hand, intake of snack foods, sweets and desserts, and sugar-sweetened beverages were associated with the presence of both outcomes. This has been documented both at the national level in Mexico as well as internationally, where the consumption of foods and drinks with high energy density, fat content, and added sugars in the place of natural foods (23, 24). These types of foods generally imply a process, and it has been argued that the marketing of these unhealthy products affects attitudes, preferences, and consumption of unhealthy foods in both children and adults, leading to excessive weight gain (25, 26).

We also found that greater fiber consumption was associated with lower overweight and obesity in school-age children, even when this association was not statistically significant. Although this association is inconclusive in the scientific literature, we suggest that a greater fiber content in food items indicates a lower caloric density, as well as a lower consumption rate and possibly a greater sense of satiety (27). Furthermore, the low consumption of fruits and vegetables in the diets of children has not generally been an area of focus, which is problematic considering the nutritional benefits of these foods and their protection against chronic diseases (28). The consumption of fruits and vegetables also assures a higher consumption of fiber, a key player in the prevention and reduction of non-transmissible conditions such as constipation: a common problem among children (29). Evidence suggests that if fruit and vegetables consumption occurs from childhood, healthy dietary habits are likely to be adopted in the long term (30).

Our results are comparable with previous studies in children which report lower indices of overweight and obesity with higher consumption of fiber (31). This may be attributable to the fact that median fiber intake is far below the recommended amount (32) for which the increase in food items high in fiber would be recommendable to strengthen this association and reduce the risk of other chronic diseases (33).

Also, as previously documented, structural variables which affirm the association of obesity with the level of general wellbeing of the school-age population are of concern. In the present study, we find that the prevalence of OB increases with increased wellbeing condition by the WBI. Previous studies have reported a wide variation in the prevalence of OW and OB in the school-age population, oscillating from 2.9 to 44.4% across different countries worldwide (34, 35), showing a considerable impact from familial socioeconomic conditions (36). Furthermore, statistically significant differences by gender have been recorded in Mexico, where the ENSANUT surveys show that across survey years the prevalence of OW and OB is higher in men (37).

This has varied implications, given the evidence that in developed countries, whose populations have experienced improvements in socioeconomic status, improvements in health have also been documented in relation to an improved access to health care. This improvement in low- and middle-income countries could mean greater mechanization, leading to lower daily activity levels and an increased access to ultra-processed foods and fast foods (38).

In children residing in rural areas, prevalence of both overweight and obesity is lower. In this sense, our results are congruent with previous results in Mexico (39) and in other Latin American countries and developing countries, where the results of gradual growth of urban areas, especially in poor urban zones (40) where migrants from rural zones tend to settle, generate changes in the conditions in which people live. These changes, linked to changes in available infrastructure, such as less movement linked to public transport and in daily activities, and in general greater sedentarism, in conjunction with changes in dietary habits (41) stemming from a greater availability of ultra-processed foods, have been linked to obesity epidemics. In this sense, each country presents a specific point in the transition toward epidemic obesity, although in general, this has initially presented itself in groups of higher socioeconomic status, and later concentrates in poorer socioeconomic groups (42).

Our study has the specific strength of its instruments being validated and used in national-level surveys in Mexico, and that anthropometric measures have been performed by trained and standardized staff. The information used is from national-level samples with the same design, which allows comparison and extrapolation of results within the national population. Furthermore, by using a pseudo-panel analysis, we could confirm that the information across various survey years was consistent and from the point of view of analysis with a lineal regression model, the possibility of bias by omission of fixed variables is lowered. This is due to the methodology which controls for unobservable heterogeneity which may be assumed as invariable over time (43), as well as the fact that the sample shows no losses across time which allows-unlike an original panel design-the number of cohorts to remain constant (44).

Among the limitations of this study is that, due to being a sequence of surveys, as well as the nature of transversal studies and the lack of continuity in public policy and related interventions, we cannot infer causality and therefore, effects. Nevertheless, we were able to observe trends over the time period of reference.

Another limitation is that the survey design does not include physical activity in children in every year that the survey was conducted, thus, it was not possible to include this variable in the pseudo-panel analysis.

Notably, when facing a problem of the magnitude of school-age child obesity, permanent government strategies and actions are required to ensure the containment, prevention, and management of this outcome in order to avoid serious consequences for long-term health and national development. To the present, various strategies have been implemented in Mexico (8, 45, 46), as well as in other countries around the world (47, 48). Although these have been associated with some positive results, they are not public policy actions which demonstrate an overall effective impact.

An example of this is the establishment of regulations on the sale and distribution of foods and drinks in the school environment dating back to 2010, and which were only put in place in 2012 and whose implementation was monitored only starting in 2015. The results of the implementation evaluation during the study years showed that only a small portion of children ate healthy food items in school. Compliance with the regulations by schools increases the probability of healthy lunches if they are bought in the school, which suggested that better implementation of standards and additional strategies are necessary to improve public policies which achieve the objective to reduce childhood obesity (49). Nevertheless, to the present this strategy has not been implemented or consolidated.

Other actions implemented on the national stage and not only targeting the school-age population include the 2014 tax of one Mexican peso on sugar-sweetened beverages (50), and one previous study found a reduction of 6% in buying of taxed beverages in 2014. Furthermore, from 2014 to 2015 changes were observed in the buying of taxed and non-taxed beverages, where buying of taxed beverages diminished by 5.5% in 2014 and 9.7% in 2015: an average reduction of 7.6% over the study period. Lower income households showed greater reductions in buying of taxed beverages during both study years (51). Buying of non-taxed beverages increased by 2.1% during the study period. Nonetheless, the tax is too small to expect the provocation of biological impacts such as a reduction in obesity. In fact, in our analysis, we observed that the consumption of sweetened beverages is so widely generalized in this population (about 90%), that the low variability increases the difficulty of associations detection.

After a difficult battle, a change was achieved in nutritional labeling in Mexico with the recently introduced frontal warning label. This was based on evidence of a national sample showing that in 2016 the most referenced label in the selection of industrialized food and drink items was the “nutritional table” at 41.5% (IC95% 36.9–46.3) of respondents, and lesser use was made of the “food label” at 4.3% (IC95% 3.1–5.7). This finding led to the proposal of a quick to read and readily understandable frontal nutritional label, which would show key nutritional criteria compliant with existing official nutritional standards recognized by the WHO, such as percent of consumption based on sugar, in an effort to address the diabetes epidemic in the country (52).

On the other hand, given the current state of global food systems, Mexico's current government launched the Intersectoral Health, Food, Environment and Competitivity Group (GISAMAC for its Spanish acronym) (53), formed by six federal departments with the participation of the Agricultural Office and sectoral bodies from each entity. Based on the guidelines of the EAT Lancet Commission (54, 55) which highlight the need for cross-cutting environmental policies which promote the availability and consumption of foods which are sustainable, affordable and healthy for the planet and therefore, for the population. Part of this included a focus on establishing within educational settings short supply chains for consumption of local foods, school gardens, food and nutrition education strategies which put children in a leading role, as well as the regulation of food and beverage marketing directed at children.

The consideration of preventative approaches to early and childhood obesity increases early detection, improves the quality of childhood care options, and offers an evidence-based communication plan for behavior change around overweight and obesity which is focused on life course and has national reach. However, the implementation and progression of this approach was stymied by the COVID-19 pandemic and is currently being retaken.

In conclusion, overweight and obesity in school-age children is a continuing problem with serious repercussions for the future. Despite carrying out a variety of individual- and global-level public policy actions for the containment, prevention, and control of this condition, structural factors exist which require early action and significant investment, especially given the integral nature of the actions needed across sectors such as health, education, economy, social development, environment, and agriculture. These actions are needed in order to modify and construct a healthy and sustainable food system in Mexico, and a healthy and sustainable diet to children.

## Data availability statement

Publicly available datasets were analyzed in this study. This data can be found at: https://ensanut.insp.mx.

## Ethics statement

The studies involving human participants were reviewed and approved by Institutional Research Ethics Committee, National Institute of Public Health of Mexico. Written informed consent to participate in this study was provided by the participants' legal guardian/next of kin.

## Author contributions

All authors listed have made a substantial, direct, and intellectual contribution to the work and approved it for publication.

## Funding

National Health and Nutrition Surveys in Mexico were funded by the Ministry of Health.

## Conflict of interest

The authors declare that the research was conducted in the absence of any commercial or financial relationships that could be construed as a potential conflict of interest.

## Publisher's note

All claims expressed in this article are solely those of the authors and do not necessarily represent those of their affiliated organizations, or those of the publisher, the editors and the reviewers. Any product that may be evaluated in this article, or claim that may be made by its manufacturer, is not guaranteed or endorsed by the publisher.
